# Assassination of President Lincoln

**Published:** 1865-04

**Authors:** 


### EDITORIAL DEPARTMENT.

### ASSASSINATION OF PRESIDENT LINCOLN.

Our Journal goes to press at the very time when the telegraph has just flashed the startling announcement that, "*President Lincoln is dead!*" Business is suspended, the bells are tolling, the entire city draped in the emblems of mourning, and every loyal heart overwhelmed with grief and sorrow; a great people were never more deeply moved. We confess inability to write upon legitimate topics, and can only think of the national loss and grief, and of the effects this tragedy will have upon the national character and life.

President Lincoln's life has been one of unsullied brightness, and throughout all time and in every country, he will be regarded as the martyred Father of American Liberty. He has given to the cause of freedom a pure and unselfish support, lived to see its triumph over slavery, and, unwarned, appeared, to receive from the great Judge, the welcome, "Well done good and faithful servant;" snatched from earthly honors and glories, to receive the congratulations of angels, and take leadership in the great army of martyred heroes.

We cannot penetrate the thick veil which separates the present from the future—the known from the unknown. We cannot understand what benevolent purpose is concealed in this providence, or fathom the mystery, why our greatest blessings should sometimes come to us in deep or even painful disguise. President Lincoln is dead—died of most foul conspiracy; but the principles which made him hated are not dead, and cannot die; they are immortal, and if they had been born to die, his death, and the death of his heroic followers, would have baptized them to an immortal life. The great principle of freedom, as opposed to slavery, is now established for all coming time; it may be that it required this crowning sacrifice. The East and the West, the North, and even the South—the whole world of civilized men will now acknowledge its rule, while the assassins and villains who deny its power, cannot escape the retributions of an incensed people, or the judgments of a just God. Changes in governmental affairs, however great, could not produce such mighty revolutions as have taken place under the leadership of President Lincoln; slavery was the rule and freedom the exception, while freedom has at last become the watchward of a regenerated country, and every additional sacrifice will only increase our love for it, while new anthems of thanksgiving and praise, shall be sung by a redeemed people. We shall not mourn for our President as "without hope." A few devils, thrice damned, will even rejoice in his death, but men of all parties will everywhere denounce the cowardly assassination, which has attempted to destroy the sick and unguarded, and in an unsuspected moment murdered its victim. Nothing has been gained, everything has been lost by the conspirators. The gain has been on the other side; freedom and human progress, have gained a worthy martyr; our army of dead, a noble leader, heaven an honest and earnest worker, and President Lincoln his eternal reward; "*but the mourners go about the streets.*"

